# Psychometric properties of the Satisfaction with Life Scale (SWLS): secondary analysis of the Mexican Health and Aging Study

**DOI:** 10.1186/s12955-016-0573-9

**Published:** 2016-12-09

**Authors:** Mariana López-Ortega, Sara Torres-Castro, Oscar Rosas-Carrasco

**Affiliations:** National Institute of Geriatrics, National Institutes of Health, Mexico, Periférico Sur 2767; San Jerónimo Lidice, México City, 10200 Mexico

**Keywords:** Psychometric properties, Satisfaction with life, Adults, Mexico

## Abstract

**Background:**

The Satisfaction with Life Scale (SWLS) has been widely used and has proven to be a valid and reliable instrument for assessing satisfaction with life in diverse population groups, however, research on satisfaction with life and validation of different measuring instruments in Mexican adults is still lacking. The objective was to evaluate the psychometric properties of the Satisfaction with Life Scale (SWLS) in a representative sample of Mexican adults.

**Methods:**

This is a methodological study to evaluate a satisfaction with life scale in a sample of 13,220 Mexican adults 50 years of age or older from the 2012 Mexican Health and Aging Study. The scale’s reliability (internal consistency) was analysed using Cronbach’s alpha and inter-item correlations. An exploratory factor analysis was also performed. Known-groups validity was evaluated comparing good-health and bad-health participants. Comorbidity, perceived financial situation, self-reported general health, depression symptoms, and social support were included to evaluate the validity between these measures and the total score of the scale using Spearman’s correlations.

**Results:**

The analysis of the scale’s reliability showed good internal consistency (α = 0.74). The exploratory factor analysis confirmed the existence of a unique factor structure that explained 54% of the variance. SWLS was related to depression, perceived health, financial situation, and social support, and these relations were all statistically significant (*P* < .01). There was significant difference in life satisfaction between the good- and bad-health groups.

**Conclusions:**

Results show good internal consistency and construct validity of the SWLS. These results are comparable with results from previous studies. Meeting the study’s objective to validate the scale, the results show that the Spanish version of the SWLS is a reliable and valid measure of satisfaction with life in the Mexican context.

## Background

Satisfaction with life is a cognitive process of judgment [1] through which individuals assess the quality of their life according to their own criteria. In this process, individuals weigh the personal priorities of their life, judging it as a whole, weighing the good against the bad, and defining it as more or less satisfactory [2]. This definition will depend on the circumstances of their life and the context that is considered appropriate [2, 3]. Life satisfaction is a component of a broader concept, subjective wellbeing, which has been studied in the field of positive psychology. The construct of subjective wellbeing has two components, one is emotional and can distinguish positive and negative affect, and a second one is a cognitive component called satisfaction with life. Thus, subjective wellbeing includes the study of happiness from the perspective of positive emotions and satisfaction with life [4, 5].

Various studies of subjective wellbeing have shown that a happy person lives longer, has better physical health, and establishes better interpersonal relationships [6, 7]. Similarly, the impact of satisfaction with life has been measured using variables such as health status, employment, economic status, and level of activity [6].

With the idea of questioning people about their life and measuring the concept of satisfaction with life, including identifying various problems with existing scales of life satisfaction, Diener and collaborators designed an instrument consisting of multiple items, the Satisfaction with Life Scale (SWLS) [1]. As noted in previous studies [8], one of the main advantages of the SWLS has been its widespread utilization. It has been used in studies consisting of diverse samples with regard to sex, age group, and occupation, to name a few, and the scale has been validated in numerous languages including Spanish [9–11], French [12], Dutch [13], Portuguese [14, 15], Turkish [8], and Chinese [16]. Currently, it is one of the most widely used scales for the measurement of wellbeing. In Spanish, validation studies and tests of psychometric properties of the SWLS have been carried out in adolescent and adult populations in Spain [11–13], Chile [17, 18], Peru [19], and Argentina [20]. Since its creation, the psychometric properties of the SWLS have shown favourable properties. The scale has shown to be a valid and reliable measure of life satisfaction, showing high internal consistency and reliability and suited for use with different age groups and populations [5]. Since then, studies with samples from many countries have confirmed these favourable properties.

Although the available information and number of studies performed regarding health have grown exponentially in Mexico, few studies have been completed at the population level regarding psychosocial wellbeing. Currently, in Mexico, the psychometric properties of the SWLS and its use in the adult population in Mexico are unknown, and only one validation study of the Life Satisfaction Index for the Third Age (LSITA-SF) using a small sample of older Mexican adults was identified [21]. The present study focuses on the evaluation of the cognitive component of subjective wellbeing. Specifically, the objective is to evaluate the psychometric properties of the SWLS in a nationally representative sample of Mexican adults.

## Methods

### Data and population

The data was obtained from the Mexican Health and Aging Study (MHAS) [22], the first prospective study of health and aging in Mexico. The baseline survey, with national and urban/rural representation of adults born in 1951 or before, was carried out in the summer of 2001, and two follow-up surveys have been conducted in 2003 and 2012. The baseline sample was drawn from the National Employment Survey (Encuesta Nacional de Empleo, ENE) where households with at least one resident aged 50 years or older were selected to be part of the sample. If more than one person was age-eligible, one was randomly selected. Where the selected respondent is married and the spouse resides in the same household, they are also recruited for the study regardless of their age [23]. All interviews are conducted in person by trained full-time interviewers of the Mexican National Statistics Institute (Instituto Nacional de Estadistica y Geografia, INEGI).

MHAS 2012 was the first to include satisfaction with life as a study outcome. The total number of interviews in 2012 consisted of 12,569 follow-up interviews from the original panel (2001 and 2003), as well as 5,896 interviews with new subjects who were representative of the cohorts born between 1952 and 1961. Interviews with completed SWLS information were selected for the study. To maintain representativeness in the study, analyses were performed on the subjects that were 50 years of age or older at the time of the interview.

### Instrument

The MHAS 2012 includes the Satisfaction with Life Scale SWLS [1] using the Spanish version made publicly available by its authors [24]. The scale’s questionnaire measures the subjective criteria of satisfaction with life on five items using multiple-choice options. The items on the scale include the following: I1. For most things, my life is close to my ideal; I2. The conditions of my life are excellent; I3. I am satisfied with my life; I4. So far, I have gotten the things that are important to me in life; and I5. If I were born again, I would change almost nothing in my life. The original SWL scale includes seven multiple-choice answers ranging from: “1 = Strongly disagree” to “7 = Strongly agree”.

Given previous experience in the creation and implementation of surveys in Mexico, regarding the possible effect of different characteristics such as age, educational attainment, and cognitive level on the individual performance of scales including previous MHAS waves, the technical-academic committee of the MHAS discussed the need of adapting the response scale of the original SWLS for use in the Mexican context. Specifically, in line with field experience and results from a review of previous studies, a large response or answer span was viewed as possibly problematic and therefore the committee decided to reduce the response options to three choices: 1 = Agree, 2 = Neither agree nor disagree, and 3 = Disagree. As each item scored from 1 to 3, the possible range of scores of the adapted SWL scale included in the MHAS is from 5 (high satisfaction) to 15 (low satisfaction).

This decision can be supported by various studies that have shown the impact of factors such as age, education level, motivation, and cognitive level of the interviewees on the performance of these scales. Mainly, they show an impact on the way the interviewed person perceives the questions and answers, possibly causing them negative perceptions such as feeling confused or bothered by the number of gradients on the scale, being unable to distinguish between different categories, or having problems understanding the format of Likert-type answer choices [25–27]. Studies have also noted thus, the need to generate adaptations to reduce the variability in the responses [27, 28].

### Statistical analysis

To analyse psychometric properties of the SWLS included in the MHAS 2012, the following analyses were performed. First, instrument reliability was assessed through the evaluation of internal consistency using Cronbach’s alpha test as well as item-test correlations. In addition, Pearson correlations among all the items on the scale (inter-item) were performed. In a second stage, construct validity was explored using three separate analysis. First, factorial evidence was estimated using Exploratory Factor Analysis (EFA). To determine if the factor analysis was appropriate for the data in the present study, prior to the EFA, the appropriateness of the data was verified using Bartlett’s test of sphericity, which ascertains the existence (or lack of existence) of dependence between items, to determine whether the variables are correlated in the population. Following Diener and collaborators [1], an exploratory factor analysis was implemented using Varimax orthogonal rotation. Second, to identify the optimum number of factors that could be extracted, a graphic representation of the eigenvalues and the number of factors was generated using the scree plot proposed by Cattell [29]. Finally, the Kaiser-Meyer-Olkin index (KMO) was estimated as a measure of the adequacy of the sample. A high internal consistency of the scale was anticipated as well as a one-dimensional structure as result of the factor analysis. A KMO measure of sampling adequacy >0.7 was defined to establish the adequacy for this data.

The third stage of the study included known-groups validity. Based in a consensus integrated by a geriatrician, a psychologist and a public health expert the total sample of the MHAS was divided in two groups, one presenting “good health conditions” and another presenting “bad health conditions”. The good-health conditions subsample included persons without any of the following conditions: diabetes, hypertension, chronic respiratory illness, heart attack, stroke, disabling pain, urgency and stress-related urinary incontinence, and painful arthritis. Those in the bad-health conditions group presented at least one of the previous conditions. Known-groups validity was evaluated by examining the difference in means of the good-health and the bad-health groups. Our hypothesis postulates that SWLS will score higher (lower satisfaction of life) in the group of “bad health conditions” than in the “good health conditions” group. The difference was determined by the Student’s *t* test.

The final stage of the analysis consisted in evaluating criterion-related validity. To do so, the scores of participants on the SWLS were compared with other constructs found in the MHAS. Specifically, perceived support from children, spouse and friends, depression symptoms, financial situation and self-rated health. Thus, comorbidity, self-reported general health, depression symptoms, perceived financial situation, and social support were included to evaluate the validity between these measures and the total score using Spearman’s correlations. Our hypothesis postulates to find a modest to moderate correlation between the total score of SWLS and other measures. All analyses were conducted with STATA statistical software [30].

## Results

### Descriptive statistics

For the definition of the study sample, cases with missing data on the SWLS were eliminated, i.e., those who did not answer the scale. Using these criteria and after having cleaned the necessary data, the final sample for the analysis consisted of 13,220 adults aged 50 years and older with data for the SWLS. Of these, 56.9% were women (*n* = 7,524) and 59.8% (*n* = 7,910) resided in urban locations with 100,000 residents or more. The mean age of the subjects who answered the SWLS was 64.7 years (SD 9.4), and in the sample, 9,188 individuals reported being married (69.5%) (Table 1).

**Table 1:** Demographic characteristics of studied population

N	13,220
Age in years, mean (SD)	64.7 (9.4)
Sex (female), n (%)	7,524 (56.9)
Marital status, n (%)	
Single, separated, or divorced	1,679 (12.7)
Married	9,188 (69.5)
Widowed	2,353 (17.8)
Education (total number of years), mean (SD)	7.4 (6.0)
Place of residence population (number of residents), n (%)	
100,000+	7,910 (59.8)
15,000–99,999	1,454 (11.0)
2,500–14,999	1,399 (10.6)
< 2,500	2,457 (18.6)

The mean score on the SWLS was 6.9 points (SD 2.3), with women yielding a slightly higher score than men, with scores of 7.0 (SD 2.4) and 6.7 (SD 2.1), respectively. The mean score and standard deviation of each of the items on the SWLS are presented in Table 2.

**Table 2:** Descriptive statistics of the Satisfaction with Life Scale scores in the studied population: mean, standard deviation and 95% confidence intervals by item of the scale

Item	Mean (SD)	95% CI
1. For most things, my life is close to my ideal	1.4 (0.7)	(1.39, 1.41)
2. The conditions of my life are excellent	1.5 (0.7)	(1.51, 1.53)
3. I am satisfied with my life	1.2 (0.5)	(1.20, 1.21)
4. So far, I have gotten the things that are important to me in life	1.3 (0.6)	(1.25, 1.27)
5. If I were born again, I would change almost nothing in my life	1.5 (0.6)	(1.52, 1.55)
Total score	6.9 (2.3)	(6.87, 6.94)

### Psychometric properties

#### Internal consistency

The analysis of the reliability of the SWLS showed an internal consistency of 0.74 (Cronbach’s alpha). Similarly, we confirmed that the inter-item correlation was significant, and all of the items had moderate to high correlations with the scale (item-test), with values in the range of 0.64 to 0.74 (Table 3).

**Table 3:** Correlations matrix and Cronbach’s Alpha for the Satisfaction with Life Scale in the studied population

Item	Item 1	Item 2	Item 3	Item 4	Item 5	Item-total Correlation	Alpha
1. For most things, my life is close to my ideal						0.68	0.71
2. The conditions of my life are excellent	0.43^a^					0.74	0.68
3. I am satisfied with my life	0.35^a^	0.45^a^				0.74	0.68
4. So far, I have gotten the things that are important to me in life	0.32^a^	0.40^a^	0.48^a^			0.72	0.69
5. If I were born again, I would change almost nothing in my life	0.27^a^	0.32^a^	0.31^a^	0.34^a^		0.64	0.73

^a^Correlation was significant at *P* < 0.001

#### Exploratory factor analysis

The determinant of the correlation matrix was 0.37, confirming an adequate fit of the matrix for the factor analysis. Bartlett’s test of sphericity (Chi-squared = 13,046.76; *P* = <0.001) showed the existence of high dependence among the five items. Meanwhile, the value of the Kaiser-Meyer-Olkin index was 0.8; and therefore, it was determined that the data were adequate to complete a factor analysis.

The exploratory factor analysis resulted in a unique factor that explained 54.2% of the total variance and had a characteristic value (eigenvalue) of 2.71. As observed in Table 4, the items showed a high saturation with factor weights varying between 0.61 for item 5 (If I were born again, I would change almost nothing in my life) and 0.83 for item 3 (I am satisfied with my life). Finally, the scree plot (Cattell) showed that the one-factor model was adequate to represent the data.

**Table 4:** Exploratory factor analysis of the 5 items of the Satisfaction with Life Scale in the studied population

Item	Factor I
1 For most things, my life is close to my ideal	0.67
2 The conditions of my life are excellent	0.76
3 I am satisfied with my life	0.83
4 So far, I have gotten the things that are important to me in life	0.78
5 If I were born again, I would change almost nothing in my life	0.61
Characteristic value (eigenvalue)	2.17
Percentage of explained variance	54.25%

Components’ matrix with Varimax orthogonal rotation

#### Known-groups validity

Mean score of the SWLS was higher (less satisfaction) in the bad-health (*n* = 12 307) group compared to the good-health group (*n* = 905) as expected, and the difference between the groups was statistically significant (*P* < .01). Mean score of the SWLS for the bad-health group was 7.0 (S.D. = 2.3) and 6.0 (S.D. =1.6) for the good-health group.

#### Validity between other measures

The correlations between the SWLS and other variables also showed the expected behaviour. Satisfaction with life correlated with: depressive symptoms [*r* =0.29 (*P* < .01) dissatisfaction increasing as depression symptoms increase]; limiting pain [*r* =0.15 (*P* < .01) those suffering pain that limits their daily activity reported lower life satisfaction], and self-reported health [*r* =0.19 (*P* < .01) those reporting poor health status reported lower life satisfaction]. In addition, in this sample of Mexican adults, life satisfaction was correlated to the respondents’ financial situation [*r* =0.23 (*P* < .01) as people report worse financial situation, lower their life satisfaction] (Table 5).

**Table 5:** Correlation of SWLS scores and selected measures

Health and economic characteristics	
Depression	0.29^a^
Limiting pain	0.15^a^
Self-reported health	0.19^a^
Financial situation	0.23^a^
Social support measures	
How much does your spouse understand your feelings about things? Would you say…A lot, little, or not at all	0.23^a^
How much can you confide in him/her if you have a serious problem? Would you say…A lot, little, or not at all	0.21^a^
How much does your spouse listen if you need to talk about your worries?	0.22^a^
How often does he/she disappoint you when you are counting on him/her?	−0.14^a^
How much do your children understand your feelings about things? Would you say…A lot, little, or not at all	0.21^a^
How much can you confide in them if you have a serious problem? Would you say…A lot, little, or not at all	0.20^a^
How much do they listen if you need to talk about your worries?	0.21^a^
How often do they disappoint you when you are counting on them?	−0.11^a^
How much do your friends, acquaintances, or colleagues at work understand your feelings about things? Would you say…A lot, little, or not at all	0.13^a^
How much can you confide in them if you have a serious problem? Would you say…A lot, little, or not at all	0.12^a^
How much do they listen if you need to talk about your worries?	0.10^a^
How often do they disappoint you when you are counting on them?	−0.07^a^

^a^Correlation was significant at *P* < 0.01

Regarding perceived social support measures, the SWLS correlated with support from spouse, children and friends, being support from the spouse the one that presented higher correlation coefficients and support from friends the lower ones (Table 5). From all the support measures, how much does the respondent perceive that his/her spouse/children/friends understand their feelings about things presented the higher coefficients with values of .23 (*P* < .01), .21 (*P* < .01) and .13 (*P* < .01), respectively (Table 5). As expected, the relation of life satisfaction with how much the respondent feels disappointed by their social network (spouse, children or friends) was negative, where individuals who feel disappointed (low score in the respective questions), were less satisfied (higher values in the scale score).

## Discussion

The main aim of this work was to present results from the first study that uses a representative sample of Mexican adults in order to validate the Satisfaction with Life Scale [1] (SWLS) in the Mexican context. This scale is a brief measure of life satisfaction validated and widely used in a variety of samples and languages across countries. This scale was included, for the first time, in the Mexican Health and Aging Study (MHAS) in 2012. MHAS is a prospective panel study with representation at the national level of adults aged 50 years and older and thus, the results can be used as a reference in future studies with samples of Mexican adults. Results from the analysis indicate that the Spanish version of the SWLS is a reliable measure of satisfaction with life in the Mexican context.

The scale’s internal consistency in this study of Mexican adults showed an acceptable reliability, producing an alpha equal to 0.74. Consistent with results from other studies, the exploratory factor analysis of the SWLS in Mexican adults showed a one-factor structure that satisfactorily explained the total variance (54%) and presented factor weights that varied between 0.611 and 0.834. These results are consistent with the factor analysis conducted by Diener et al. [1] when creating the original instrument, which resulted in a single factor structure explaining 66% of the variance and a factor weight that ranged between 0.61 and 0.84, results that have been replicated in various studies [4, 5, 9, 13, 15, 31]. As mentioned in previous studies, it is of great relevance that the results of the present study have been replicated in numerous previous studies, independent of the country and language in which the scale is applied, reinforcing its validity in the Mexican context.

In a study of older Spanish women who remain active, Requena Hernández and collaborators [10] found a single factor that explained 51.3% of the variance. For adults 18 to 65 years of age in Chile, Vera-Villaroel and collaborators [18] found a one-factor structure that explained 59.3% of the variance and the saturation of this factor was between 0.56 and 0.88.

For this Mexican sample, the results show that the scale has good internal consistency and favourable factor validity, and the results are similar to those found in the original study, and studies using the SWLS in diverse languages and populations samples. Among others, the present study shows similar results as those validated in university students and older adults [1, 11], to results in different countries that used the original scale in English, or translated it to other languages [9, 13, 15, 18], where high internal consistency was found within ranges between .79 and .80 (Cronbach’s alpha).

Satisfaction with life in this Mexican sample was related to depression, limiting pain, self-reported health, financial situation, and social support, and these relations ranged from *r* = 0.15 to *r* = 0.29 and were all statistically significant (*P* < .01), although these correlations were modest, they confirm the results found in studies with samples from different countries [2, 4, 10, 12]. The main measure correlated with SWLS was depression symptoms (loss of self-worth, sadness, feelings of despair, feelings of hopelessness or helplessness between others) can interfere directly with the perception of satisfaction of life at the same time, previous studies have found similar results [2, 15]. The fact that perceived social and family support was significantly related to life satisfaction in this study with Mexican adults corroborates findings of previous studies showing how perceived social support has an important role in defining adult and older adult’s individual psychological well-being.

This study is considered relevant as it is the first study to validate the SWLS scale in a representative sample of Mexican adults and older adults, however, there are some limitations to the study. First, the modified scale including 3 response options versus the original with 7 options, could affect the total score and therefore the cutoffs proposed by Diener and colleagues are not applicable. Therefore, this scale can only be used based on the total score (continuously) and the three answer options used to categorise participants and be useful to perform in large surveys as performed in this study. Despite of this change, the Cronbach’s alpha remained acceptable and the one-factor structure was found similar to other studies as previously mentioned. Second, the results of this study could be of use for other similar contexts in Spanish speaking countries, however, while in the Mexican case this was not the case, care should be taken as cultural language differences may be of concern in the use of the original Spanish translation made available by its authors, possible needing further adaptation. Finally, the fact that we were not able to examine other psychometric properties of the scale through procedures such as test–retest reliability and inter-observer agreement to further test the validity of the scale, or conduct a test of convergent validity given no other life satisfaction components were included in the MHAS are also limitations of the study. Finally, while for this study predictive validity of life satisfaction for adverse outcomes such as use of health services and mortality could not be performed given this was the first wave of the MHAS to include the SWLS, this analysis should be complemented in the future using new waves of the survey.

## Conclusions

Overall, this study validating the SWLS in a representative sample of adults 50 years and older found that the scale shows internal consistency, favourable factor validity, and acceptable criterion-validity. Thus, its use can be recommended in other samples of adults aged 50 years and older in the country as well as in countries of similar economic and cultural backgrounds. In addition, this validation shows the potential utility in national surveys, clinical practice and research in other groups or contexts in Mexico as it has been noted in previous studies [8, 14]. Given the important role of life satisfaction in overall psychological well-being, we support previous work that emphasises how besides illness and disability, health-care systems should be concerned and working towards supporting methods to improve positive psychological states [32].
